# Complete resection of a recurrent bronchogenic cyst tightly adhered to the left atrium using cardiopulmonary bypass: a case report

**DOI:** 10.1186/s44215-024-00140-y

**Published:** 2024-02-21

**Authors:** Tomonori Furugen, Takao Teruya, Shoko Nakasone, Hidenori Kawasaki, Masahiro Toyama, Keita Miyaishi, Syotaro Higa, Mizuki Ando, Tatsuya Maeda, Yuya Kise, Hitoshi Inafuku, Moriyasu Nakaema, Takaaki Nagano, Kojiro Furukawa

**Affiliations:** 1 Department of Thoracic and Cardiovascular Surgery, Graduate School of Medicine, University of the Ryukyus, 207 Uehara, Nishihara, Okinawa, 903-0215 Japan; 2 Department of Surgery, NHO Okinawa Hospital, 3-20-14 Ganeko, Ginowan, Okinawa, 901-2214 Japan

**Keywords:** Recurrent mediastinal bronchogenic cyst, Complete resection, Cardiopulmonary bypass

## Abstract

**Background:**

Most surgeons agree that symptomatic mediastinal bronchogenic cysts should be resected, and complete resection is considered mandatory to avoid recurrence. However, a symptomatic mediastinal bronchogenic cyst sometimes adheres to a vital organ, making complete resection hazardous. In such case, surgical resection using cardiopulmonary bypass should be performed to achieve complete resection.

**Case presentation:**

A 65-year-old female patient had undergone surgical drainage of a mediastinal bronchogenic cyst 30 years ago. Recently, she presented with complaints of orthopnea, and computed tomography revealed a few days later a subcarinal bronchogenic cyst markedly compressing the left atrium. Complete resection of the mediastinal bronchogenic cyst was performed using cardiopulmonary bypass. The postoperative course was uneventful. The patient was followed up for 1 year, with no recurrence of the bronchogenic cyst.

**Conclusions:**

For patients with mediastinal bronchogenic cysts compressing the left atrium, we recommend complete resection using cardiopulmonary bypass.

## Background

Bronchogenic cysts (BC) are a congenital abnormality. Although some cysts are asymptomatic, they may cause cardiac failure when compressing the left atrium (LA), albeit rarely [1]. Most surgeons agree that symptomatic BC should be completely resected. However, symptomatic BC may result in the formation of dense adhesions between surrounding vital structures, precluding a complete resection [2]. Herein, we report a case of BC compressing the LA that was completely resected using cardiopulmonary bypass (CPB).

## Case presentation

**Fig. 1:**
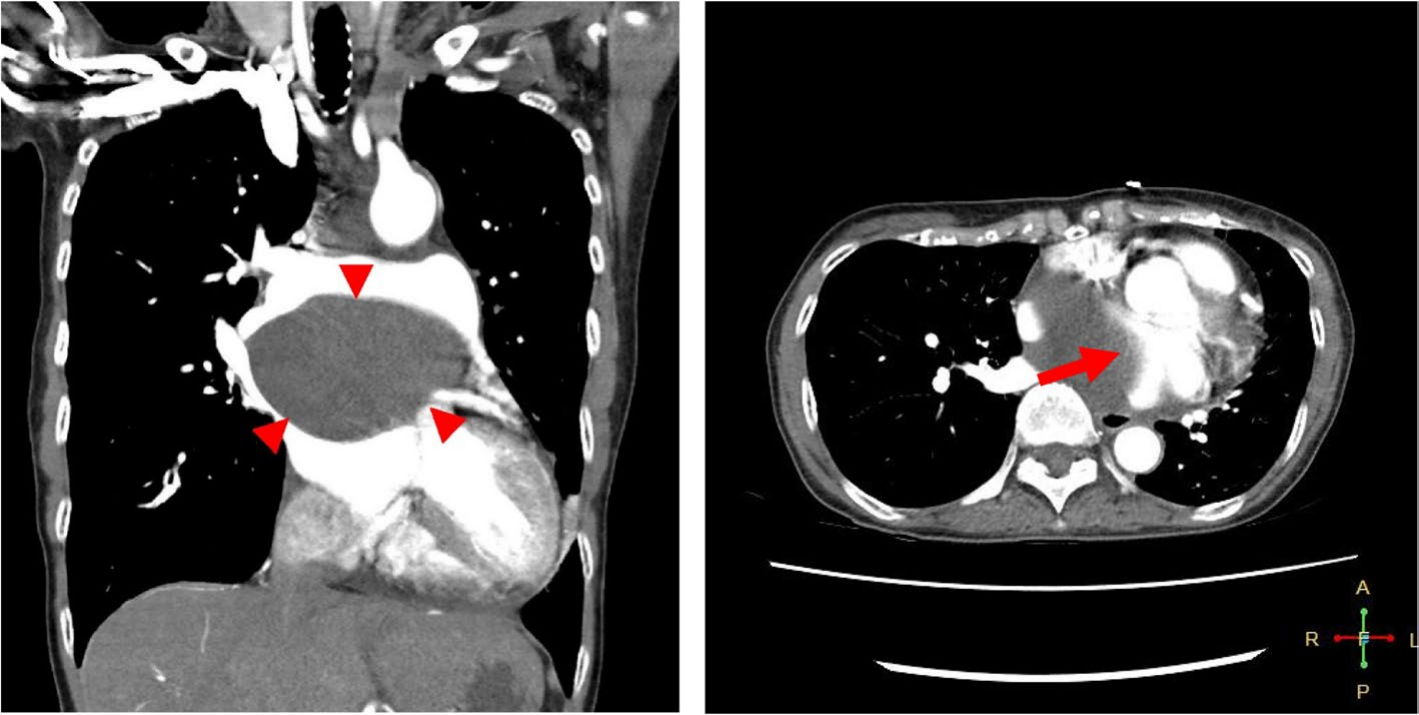
Chest computed tomography images: The cystic lesion (arrowhead) is located between the right main pulmonary artery and the left atrium. The cystic wall strongly adheres to the LA (arrows)

A 65-year-old female had undergone surgical drainage and alcohol ablation therapy for asymptomatic mediastinal BC 30 years ago. The resected specimen was histologically diagnosed as BC. In the meantime, the patient did not complain of any symptoms. Subsequently, the management approach involved watchful waiting at a different hospital. However, after recent complaints of orthopnea, the patient was admitted to another hospital. Electrocardiography revealed paroxysmal atrial tachycardia. Contrast-enhanced chest computed tomography (CT) revealed a solitary, well-defined, low-density mass measuring 11 × 10 cm in the subcarinal space (Fig. 1). The mass also displaced the LA. Transthoracic echocardiography revealed moderate systolic dysfunction, with a left ventricular ejection fraction (LVEF) of 41%. No obvious pericardial effusion was observed. The previous hospital team devised a treatment plan, comprising emergency transbronchial needle drainage followed by elective resection via thoracotomy. Upon exploration, the team observed that the BC was adherent to the left main bronchus; hence, dissection of the subcarinal cyst resulted in injury to the left main bronchus. Complete resection was considered high-risk, consequently, surgical drainage was repeated. Subsequently, the patient’s condition improved. However, a month later, she complained of similar symptoms that progressively worsened. Follow-up CT revealed a large cyst compressing the LA. BC recurrence was identified, and the patient was referred to our hospital.

**Fig. 2:**
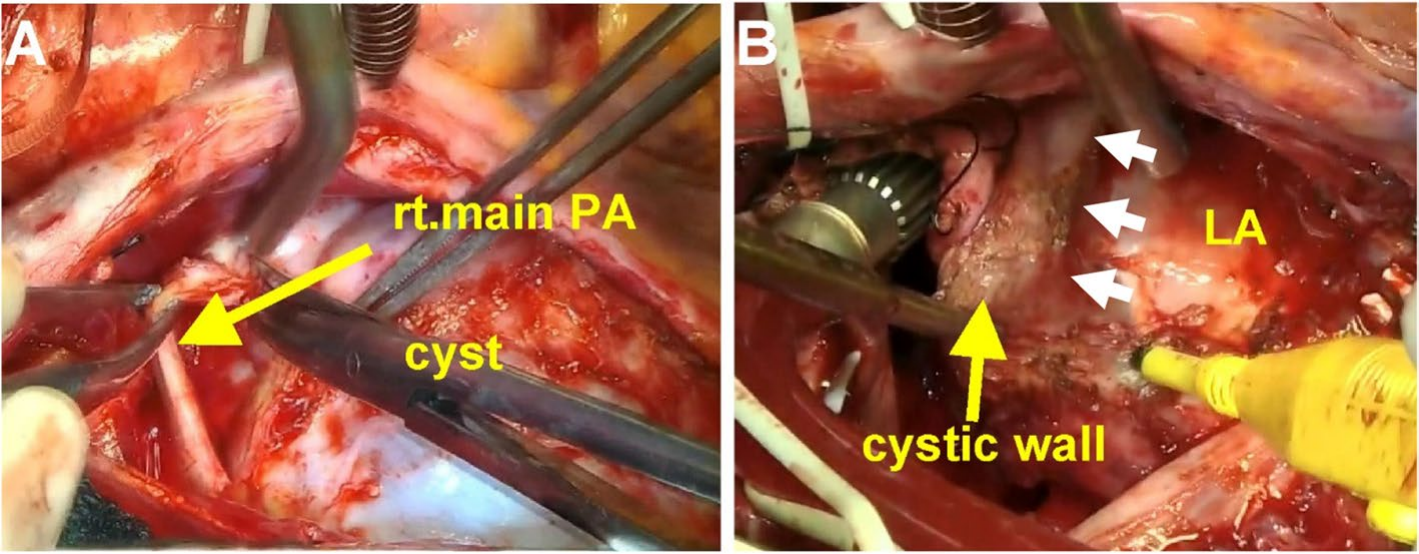
Operative view. **A** The cyst was dissected from the right main pulmonary artery (rt. main PA). **B** The cystic wall was dissected from the left atrium (LA). The cystic wall strongly adheres to the LA (white arrows)

We examined the patient and determined that she was a good candidate for the resection of cystic lesions using CPB. As orthopnea was exacerbated in the supine position, anesthesia was induced using extracorporeal membrane oxygenation. Sternotomy with CPB support was performed to expose the BC, which was located between the ascending aorta and the superior vena cava. Dissection of the pulmonary artery (PA) was performed easily (Fig. 2A). However, the BC was firmly attached to the LA and left the main bronchus. To better expose the deep anatomical structures, the BC was evacuated intraoperatively via needle aspiration while ensuring that the fluid culture remained sterile. Dense adhesions impeded access to the LA; therefore, aortic cross-clamping and cardiac arrest with cardioplegic protection were necessary. The BC wall was safely resected along the roof of the LA (Fig. 2B), and a complete resection was performed.

**Fig. 3:**
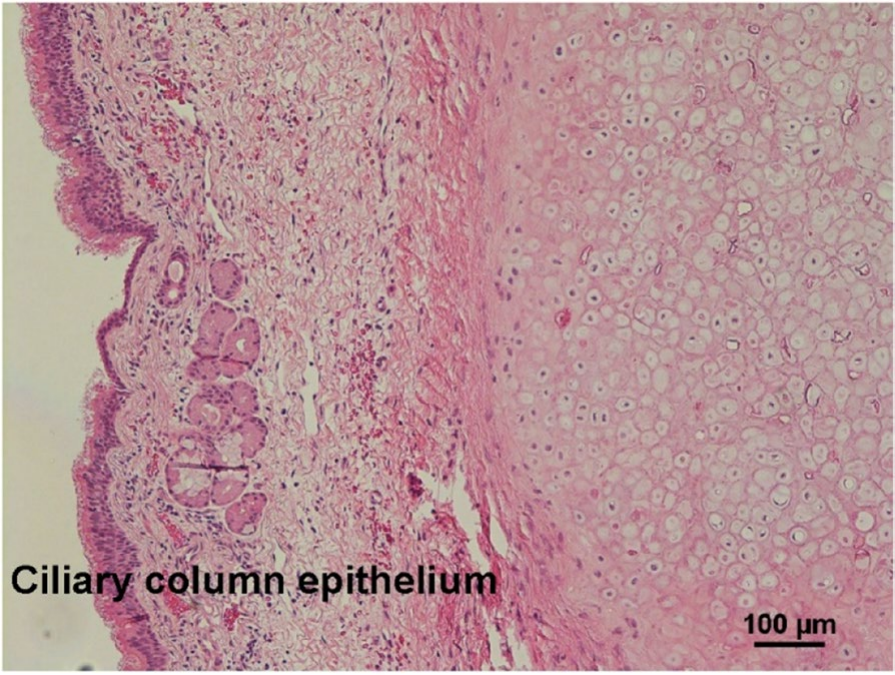
Histological image (hematoxylin-eosin stain × 40) revealed a ciliated columnar lining

The postoperative course was uneventful. Histological examination revealed a ciliated columnar lining, which confirmed that the lesion was BC (Fig. 3).

**Fig. 4:**
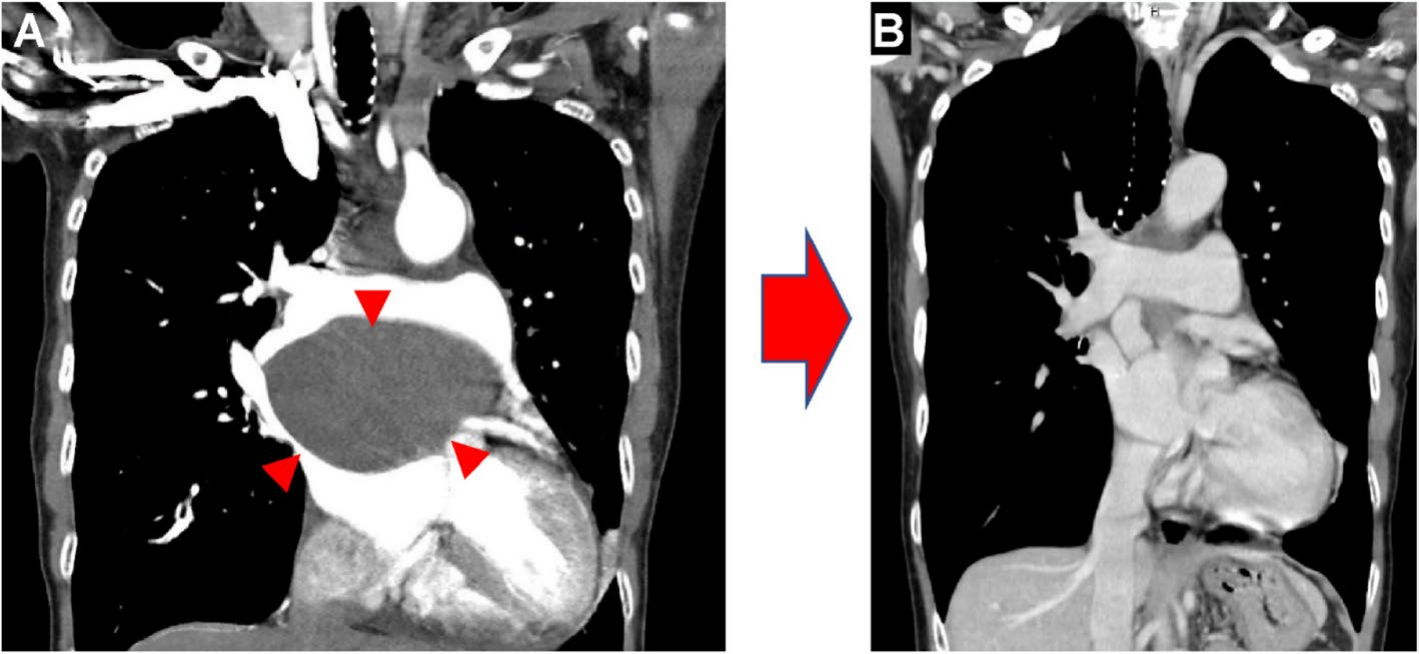
Chest CT images. **A** The cystic lesion (arrowhead) is encountered preoperatively. **B** The cystic lesion is completely resected postoperatively

On outpatient follow-up a year later, the patient denied any symptoms. Follow-up CT revealed no cysts. Furthermore, the right main PA and LA were not compressed (Fig. 4). Follow-up echocardiography showed recovery of cardiac function, as indicated by a postoperative LVEF of 52%.

## Discussion and conclusions

BC is a congenital disorder that occurs in the ventral foregut during embryogenesis. Several classifications of mediastinal BC have been proposed by Maier, namely, paratracheal, carinal, hilar, paraesophageal, and miscellaneous. In a previous study, mediastinal BC was found to be symptomatic in 44 of the 69 patients (63.7%) examined [3]. To the best of our knowledge, only two reports have described mediastinal BC compressing the heart, resulting in cardiac dysfunction [1, 4].

There is a consensus in favor of surgery for symptomatic BC. One case report described the successful excision of a giant subcarinal BC using video-assisted thoracoscopic surgery (VATS) [5]. However, another study reported catastrophic complications in BC resection using VATS [6]. During thoracotomy, the cyst firmly adhered to the LA. Dissecting the cyst from the LA caused lethal bleeding, and the patient was lost. Moreover, another report described incomplete resection in 11 of 48 surgical cases (23%) of symptomatic BC [7].

CPB is useful for ensuring adequate visualization and complete resection when performing complex mediastinal tumor resections. A recent case series described that in another mediastinal tumor, such as an immature teratoma, eight patients were resected with CPB, with an operative mortality of zero [8]. Moreover, seven of eight (87%) cases were resected with CPB during intrapericardial and intracardiac BC resection [4]. To the best of our knowledge, only a few cases of complete resection with CPB have been described, to be successfully performed for intrapericardial BC associated with congestive heart failure [4, 9]. Several complications may occur owing to CPB, with bleeding related to systemic heparinization being a major concern [10]. While complete resection is not always necessary in this disease, in our case, it was conducted with CPB to prevent the recurrence of mediastinal BC. For these reasons, in cases of BC tightly adherent to the LA, we advocate the use of CPB for adequate visualization and complete resection.

## Data Availability

The datasets used and/or analyzed in the current study are available from the corresponding author upon reasonable request.

## Abbreviations

BC: Bronchogenic cysts
CPB: Cardiopulmonary bypass
CT: Computed tomography
LA: Left atrium
LVEF: Left ventricular ejection fraction
PA: Pulmonary artery
EBUS-TBNA: Endobronchial ultrasound-guided transbronchial needle aspiration
VATS: Video-assisted thoracoscopic surgery
